# Skeletal muscle area predicts the outcomes of non-small-cell lung cancer after trimodality therapy

**DOI:** 10.1093/icvts/ivad020

**Published:** 2023-01-25

**Authors:** Kenji Watanabe, Fumihiko Kinoshita, Tomoyoshi Takenaka, Taichi Nagano, Yuka Oku, Keisuke Kosai, Yuki Ono, Naoki Haratake, Mikihiro Kohno, Takeshi Kamitani, Tadamasa Yoshitake, Tatsuro Okamoto, Mototsugu Shimokawa, Kousei Ishigami, Tomoharu Yoshizumi

**Affiliations:** Department of Surgery and Science, Graduate School of Medical Sciences, Kyushu University, Fukuoka, Japan; Department of Thoracic Oncology, National Hospital Organization Kyushu Cancer Center, Fukuoka, Japan; Department of Surgery and Science, Graduate School of Medical Sciences, Kyushu University, Fukuoka, Japan; Department of Surgery and Science, Graduate School of Medical Sciences, Kyushu University, Fukuoka, Japan; Department of Surgery and Science, Graduate School of Medical Sciences, Kyushu University, Fukuoka, Japan; Department of Surgery and Science, Graduate School of Medical Sciences, Kyushu University, Fukuoka, Japan; Department of Surgery and Science, Graduate School of Medical Sciences, Kyushu University, Fukuoka, Japan; Department of Surgery and Science, Graduate School of Medical Sciences, Kyushu University, Fukuoka, Japan; Department of Surgery and Science, Graduate School of Medical Sciences, Kyushu University, Fukuoka, Japan; Department of Clinical Radiology, Graduate School of Medical Sciences, Kyushu University, Fukuoka, Japan; Department of Clinical Radiology, Graduate School of Medical Sciences, Kyushu University, Fukuoka, Japan; Department of Thoracic Oncology, National Hospital Organization Kyushu Cancer Center, Fukuoka, Japan; Department of Biostatistics, Yamaguchi University Graduate School of Medicine, Yamaguchi, Japan; Department of Clinical Radiology, Graduate School of Medical Sciences, Kyushu University, Fukuoka, Japan; Department of Surgery and Science, Graduate School of Medical Sciences, Kyushu University, Fukuoka, Japan

**Keywords:** Lung cancer, Neoadjuvant chemoradiotherapy, Sarcopenia, Skeletal muscle

## Abstract

**OBJECTIVES:**

Sarcopenia correlates with poor prognosis in various malignancies. However, the prognostic significance of sarcopenia remains to be determined in patients with non-small-cell lung cancer who undergo surgery after receiving neoadjuvant chemoradiotherapy (NACRT).

**METHODS:**

We retrospectively reviewed the patients with stage II/III non-small-cell lung cancer who underwent surgery following NACRT. The paravertebral skeletal muscle area (SMA) (cm^2^) at the 12th thoracic vertebra level was measured. We calculated the SMA index (SMAI) as SMA/squared height (cm^2^/m^2^). Patients were divided into low and high SMAI groups, and the association of SMAI with clinicopathological factors and prognosis was assessed.

**RESULTS:**

The patients’ [men, 86 (81.1%)] median age was 63 (21–76) years. There were 106 patients including 2 (1.9%), 10 (9.4%), 74 (69.8%), 19 (17.9%) and 1 (0.9%) patients with stage IIA, IIB, IIIA, IIIB and IIIC, respectively. Of the patients, 39 (36.8%) and 67 (63.2%) were classified in the low and the high SMAI groups, respectively. Kaplan–Meier analysis showed that the low group had a significantly shorter overall survival and disease-free survival than the high group. Multivariable analysis identified low SMAI as an independent poor prognostic factor for overall survival.

**CONCLUSIONS:**

Pre-NACRT SMAI correlates with poor prognosis; therefore, assessing sarcopenia based on pre-NACRT SMAI may help determine optimal treatment strategies and suitable nutritional and exercise interventions.

**Figure:**
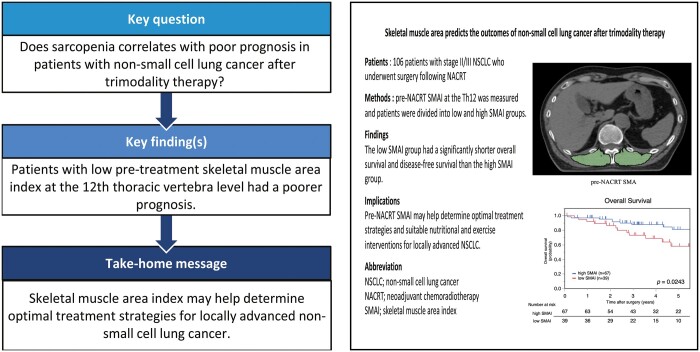

## INTRODUCTION

Lung cancer continues to be one of the most common leading causes of cancer-related death worldwide [1]. Cases of locally advanced non-small-cell lung cancer (NSCLC) constituted ∼31% of the cases reported in the 8th edition of the TNM classification of malignant tumours [2]. Locally advanced NSCLC, particularly mediastinal lymph node metastasis-positive stage IIIA-N2 cancer, is a heterogeneous disease characterized by anatomically locally advanced disease with latent micrometastases. Thus, surgical resection or radiotherapy alone is ineffective in treating this disease. Nonetheless, locally advanced NSCLC may be treated with surgery following neoadjuvant chemoradiotherapy (NACRT). The inter-group trial 0139 demonstrated that patients treated with NACRT plus surgery exhibit better progression-free survival than those treated with definitive chemoradiotherapy alone (median progression-free survival, 12.8 vs 10.5 months; hazard ratio, 0.77; 95% confidence interval 0.62–0.96; *P *=* *0.017), with no significant difference in overall survival (OS) between the 2 groups (median OS 23.6 vs 22.2 months; hazard ratio 0.87; 95% confidence interval 0.70–1.10; *P *=* *0.24) [3]. Therefore, effective prognostic and therapeutic strategies must be urgently developed to improve patient outcomes in locally advanced NSCLC.

Sarcopenia is characterized by low levels of the following 3 parameters: (i) muscle strength, (ii) muscle quantity/quality and (iii) physical performance as a severity indicator [4]. The cross-sectional skeletal muscle area (SMA) derived from computed tomography (CT) scans correlates with sarcopenia in patients with cancer. Low SMA is reportedly a poor prognostic factor in patients with early-stage NSCLC who underwent surgery [5] as well as in those with advanced NSCLC who received chemotherapy or anti-PD-1 inhibitors [6, 7].

Limited studies have focused on the association between prognosis and sarcopenia in patients with NSCLC who underwent surgery after receiving NACRT. Therefore, this study sought to determine the prognostic significance of SMA index (SMAI) in patients with locally advanced stage II/III NSCLC who underwent lung resection following NACRT.

## PATIENTS AND METHODS

### Ethical statement

This study was reviewed and approved by our Institutional Review Board (Kyushu University, Japan; IRB No. 2020-807 on 31 March 2021), and in compliance with the patient informed consent regulations. Written informed consent was obtained from each patient according to the regulations of each participating institution.

### Patients and treatment

We retrospectively reviewed the data of consecutive patients with locally advanced stage II/III NSCLC who underwent lung resection after receiving NACRT between January 2010 and December 2020 at the Department of Surgery and Science, Graduate School of Medical Sciences, Kyushu University, Fukuoka, Japan, and the Department of Thoracic Oncology, National Hospital Organization, Kyushu Cancer Center, Fukuoka, Japan. This study included patients whose pre-NACRT digital CT scans of the 12th thoracic vertebra (Th12) were available in the image archive and communication systems. Clinical and pathological tumour stages were determined based on the TNM classification, 8th edition [2]. The following patient characteristics and outcomes were recorded: sex, age, body mass index (BMI), smoking history (pack-year index), forced expiratory volume in 1 s, surgical procedure, perioperative outcomes and histopathological findings. The indication for NACRT and surgery as well as the chemotherapy regimen and radiotherapy specifics were determined through a consensus of board-certified respiratory physicians, thoracic surgeons, and radiation oncologists. The chemotherapy regimens primarily involved cisplatin in combination with tegafur/gimeracil/oteracil (S-1). All patients received three-dimensional conformal radiotherapy, as described previously [8]. The gross tumour contained the primary tumour as well as the clinically diagnosed metastatic lymph node. After surgery, the patients underwent routine follow-up, which included physical examinations, blood tests and chest radiography, every 3 months for the 1st 3 years and every 6 months thereafter. There were no missing values for the items analysed in this study.

### Imaging and assessment of skeletal muscle area

In this study, SMA was assessed using a method similar to that previously reported by our institution [9]. Pre-NACRT CT scans were performed within 1 month before NACRT. Paravertebral SMA (cm^2^) was measured at the Th12 level using the OsiriX Lite software (32-bit, version 12.5; OsiriX Lite, Pixmeo SARL Bernex, Switzerland) with a threshold of −29 to 150 Hounsfield units [10]. The muscle areas that were not clearly targeted were deleted manually. The SMAI was defined as the paravertebral SMA (cm^2^) at the Th12 level divided by the squared height (m^2^), as reported previously [9].

### Statistical analyses

The Shapiro–Wilk normality test was performed to determine whether pre-NACRT SMAI was normally distributed. The cut-off values of pre-NACRT SMAI for men and women were determined using receiver operating characteristic (ROC) curves for OS, as reported previously [11]. The cut-off values of pre-NACRT SMAI for men and women were set at 11.6 and 10.8 cm^2^/m^2^, respectively. Patient characteristics were analysed using Fisher’s exact and Student’s *t*-tests. Disease-free survival (DFS) was defined as the time between surgery and the date of recurrence or death from any cause; OS was defined as the time between surgery and the date of death from any cause. We assessed the proportional hazard propensity in the OS for the SMAI to determine whether or not the proportional risk assumption was met. The analyses were performed using the Kaplan–Meier method with log-rank test. The association of DFS and OS with clinicopathological variables was assessed using log-rank tests in univariable analysis and Cox proportional hazards models in multivariable analysis. The multivariable analysis in this study was performed using backward stepwise selection: all factors listed in the table were tested, and factors with little prognostic impact (i.e. those with large *P*-values) were excluded until all factors had *P*-values < 0.05. Finally, the remaining factors were listed as independent prognostic factors. The *P*-values of <0.05 were considered statistically significant. All statistical analyses were performed using JMP 15.0 (SAS Institute, Cary, NC, USA).

## RESULTS

### Patient characteristics

This study included a total of 106 patients with NSCLC who underwent surgical resection after receiving NACRT (Tables 1 and 2). The median age of the cohort was 63 years [interquartile range (IQR), 57–67 years]; of the patients, 86 (81.1%) were men and 91 (85.8%) had a history of smoking (median pack-year index, 42; IQR, 20–60). Before receiving NACRT, the median BMI was 23.0 kg/m^2^ (IQR, 21.0–24.3). The median dose of radiation administered was 40 (IQR, 40–40) Gy. The chemotherapy regimens varied, but all patients received chemotherapy with platinum-containing agents such as cisplatin and carboplatin. A total of 89 patients received cisplatin-based chemotherapy, whereas the others received a carboplatin-based chemotherapy. Furthermore, 20 patients (19%) received a reduced dose of chemotherapy. No treatment-related deaths were observed in our cohort (Table 1). There were 2 (1.9%), 10 (9.4%), 74 (69.8%), 19 (17.9%) and 1 (0.9%) patients with pre-NACRT stage IIA, IIB, IIIA, IIIB and IIIC NSCLC, respectively. A total of 17 patients (16.0%) underwent pneumonectomy and 89 (84.0%) underwent lobectomy or bilobectomy (Table 2). Patients were diagnosed with the following histological types: 63 (59.4%) patients with adenocarcinoma, 34 (32.1%) with squamous cell carcinoma and 9 (8.5%) with other histological types. The number of recurrence, cancer-specific death and other-cause death events were 47 (44.3%), 18 (17.0%) and 6 (0.6%), respectively.

**Table 1:** Preoperative characteristics of the cohort

Characteristics	
Age (years), median (range)	63 (21–76)
Sex, *n* (%)	
Female	20 (18.9)
Male	86 (81.1)
Smoking, *n* (%)	
Never smoker	15 (14.2)
Smoker	91 (85.8)
Pack year index, median (range)	42 (0–153)
cT factor, *n* (%)	
1	19 (17.9)
2	35 (33.0)
3	27 (25.5)
4	25 (23.6)
cN factor, *n* (%)	
0	16 (15.1)
1	16 (15.1)
2	72 (67.9)
3	2 (1.9)
cStage, *n* (%)	
IIA	2 (1.9)
IIB	10 (9.4)
IIIA	74 (69.8)
IIIB	19 (17.9)
IIIC	1 (0.9)
FEV1.0%, *n* (%)	
<70%	39 (36.8)
>70%	67 (63.2)
BMI (kg/m^2^), median (range)	23.0 (15.9–35.2)
Chemotherapy, *n* (%)	
Standard	86 (81.1)
Cisplatin + S-1	59 (55.7)
Carboplatin + paclitaxel	15 (14.2)
Cisplatin + vinorelbine	11 (10.4)
Cisplatin + etoposide	1 (0.9)
Reduced	20 (18.9)
Cisplatin + S-1	16 (15.1)
Carboplatin + paclitaxel	3 (2.8)
Cisplatin + vinorelbine	1 (0.9)
Radiation dose (Gy), median (range)	40 (40–60)

BMI: body mass index; cN: clinical N; cStage: clinical stage; cT: clinical T; FEV1: forced expiratory volume in 1 s; S-1: tegafur/gimeracil/oteracil.

**Table 2:** Perioperative characteristics of the cohort

Characteristics	
Surgical procedure, *n* (%)	
Lobectomy/bilobectomy	89 (84.0)
Pneumonectomy	17 (16.0)
Perioperative bleeding (ml), median (range)	179 (10–3860)
Operating time (min), median (range)	349 (197–952)
Concomitant resection, *n* (%)	
Present	40 (37.7)
Absent	66 (62.3)
pT factor, *n* (%)	
0	16 (15.1)
1	37 (34.9)
2	29 (27.4)
3	16 (15.1)
4	8 (7.5)
pN factor, *n* (%)	
0	66 (62.3)
1	15 (14.2)
2	20 (18.9)
pStage, *n* (%)	
0	13 (12.3)
I	32 (30.2)
II	30 (28.3)
III	31 (29.2)
Histological type, *n* (%)	
Adenocarcinoma	63 (59.4)
Squamous cell carcinoma	34 (32.1)
Others	9 (8.5)
Complications, *n* (%)	
Present	24 (22.6)
Absent	82 (77.4)
Chest tube duration (days), mean (range)	4.11 (1–29)
Postoperative hospitalizations (days), mean (range)	19.2 (7–124)

pN: pathological N; pStage: pathological stage; pT: pathological T.

### Pre-neoadjuvant chemoradiotherapy skeletal muscle area

**Figure 1:**
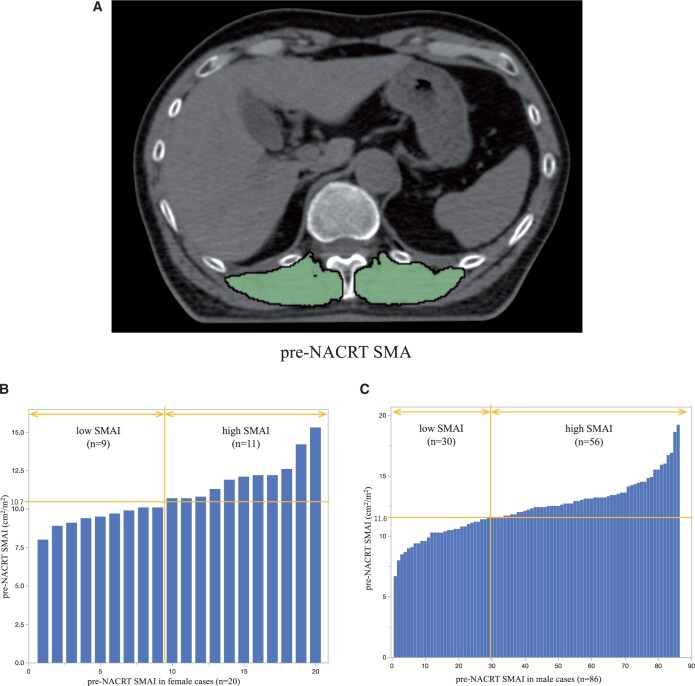
(**A**) Paravertebral muscle area at the 12th thoracic vertebra level before NACRT (example). Green highlights indicate the skeletal muscle area. (**B**) Histogram of SMAI in female patients. (**C**) Histogram of SMAI in male patients. NACRT: neoadjuvant chemoradiotherapy; SMAI: skeletal muscle area index.

Figure 1A presents a representative CT scan performed before NACRT. The median pre-NACRT Th12 SMAI was 12.40 (IQR 10.82–13.34) cm^2^/m^2^ for men and 10.69 (IQR 9.56–12.14) cm^2^/m^2^ for women. The Shapiro–Wilk normality test was performed, and the *P*-value was 0.1774, indicating that the SMAI was normally distributed. Based on the ROC curves for OS, the cut-off values of pre-NACRT SMAI for men and women were set at 11.6 and 10.7 cm^2^/m^2^, respectively. The area under curves (AUC) of ROC curves for men and women were 0.513 and 0.667, respectively. Accordingly, 39 (36.8%) and 67 (63.2%) patients were classified into low SMAI and high SMAI groups, respectively (Fig. 1B and C).

### Association between skeletal muscle area index and clinicopathological characteristics

Table 3 shows the association between the clinicopathological factors and SMAI. Fisher’s exact test showed that, among patients with a low SMAI, there were significantly more patients with a low pre-NACRT BMI (*P *<* *0.001) and excessive perioperative bleeding (*P *=* *0.025) than patients with a high SMAI.

**Table 3:** Association between skeletal muscle area index and clinicopathological characteristics of the cohort

Characteristics	Low SMAI (*n* = 39)	High SMAI (*n* = 67)	*P*-Value
Age (years), median (range)	65 (43–76)	62 (21–75)	0.085
Sex, *n* (%)			
Female	9 (23.1)	11 (16.4)	0.40
Male	30 (76.9)	56 (83.6)	
Smoking, *n* (%)			
Never smoker	8 (20.5)	7 (10.4)	0.162
Smoker	31 (79.5)	60 (89.6)	
Pack year index, median (range)	42 (0–132)	42 (0–153)	0.089
cStage, *n* (%)			
IIA	1 (2.6)	1 (1.5)	0.53
IIB	3 (7.7)	7 (10.5)	
IIIA	29 (74.5)	45 (67.2)	
IIIB	5 (12.8)	14 (20.9)	
IIIC	1 (2.6)	0 (0)	
FEV1.0%, *n* (%)			
<70%	13 (33.3)	26 (38.8)	0.68
>70%	26 (66.7)	41 (61.2)	
BMI (kg/m^2^), median (range)	21.2 (15.9–26.9)	22.2 (17.7–35.2)	<0.001
Chemotherapy, *n* (%)			
Standard	32 (82.1)	54 (80.6)	>0.99
Reduced	7 (17.9)	13 (19.4)	
Radiation dose (Gy), median (range)	30 (40–60)	40 (40–66)	0.053
Surgical procedure, *n* (%)			
Lobectomy/bilobectomy	33 (84.6)	56 (83.6)	>0.99
Pneumonectomy	6 (15.4)	11 (16.4)	
Perioperative bleeding (ml), median (range)	200 (10–3860)	170 (20–1048)	0.025
Operating time (min), median (range)	328 (197–952)	355 (207–644)	0.45
Concomitant resection, *n* (%)			
Present	16 (41.0)	24 (35.8)	0.68
Absent	23 (59.0)	43 (64.2)	
pStage, *n* (%)			
0	4 (10.3)	9 (13.4)	0.81
I	11 (28.2)	21 (31.3)	
II	11 (28.2)	19 (28.4)	
III	13 (33.3)	18 (26.9)	
Histological type, *n* (%)			
Adenocarcinoma	24 (61.5)	39 (58.2)	0.64
Squamous cell carcinoma	13 (33.3)	21 (31.3)	
Others	2 (5.1)	7 (10.4)	
Complications, *n* (%)			
Present	11 (28.2)	13 (19.4)	0.34
Absent	28 (71.8)	54 (80.6)	
Chest tube duration (days), mean (range)	4.82 (1–29)	3.70 (1–9)	0.11
Postoperative hospitalizations (days), mean (range)	19.3 (8–72)	19.2 (7–124)	0.99

BMI: body mass index; cStage: clinical stage; FEV1: forced expiratory volume in 1 s; SMAI: skeletal muscle area index.

### Prognostic effect of skeletal muscle area index on survival and recurrence

**Figure 2:**
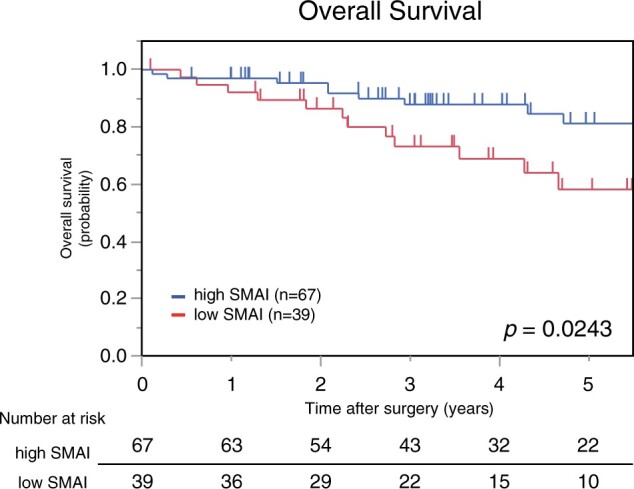
Kaplan–Meier curves for overall survival based on SMAI. The low SMAI group had a significantly shorter overall survival than the high SMAI group. SMAI: skeletal muscle area index.

The prognostic effects of SMAI on survival and recurrence were assessed using the Kaplan–Meier method. Patients with a low SMAI had a significantly shorter OS than those with a high SMAI (5-year OS, 58% vs 81%; *P *=* *0.024; Fig. 2). Furthermore, patients with a low SMAI had a significantly shorter DFS than those with a high SMAI (*P *=* *0.042). The Kaplan–Meier curves are shown in Supplementary Material, Fig. S1.

We also assessed the proportional hazard propensity in the OS for the SMAI. The result of the test of proportional hazard property was *P *=* *0.96, and we conclude that the proportional risk assumption was met.

The results of the univariable and multivariable analyses of the clinicopathologic factors related to OS are shown in Table 4. In the univariable analysis, age (≥70 vs <70 years; *P *=* *0.040) and pre-NACRT SMAI (low versus high; *P *=* *0.029) were found to be significant prognostic factors associated with OS, whereas in the multivariable analysis, the independent prognostic factors for OS were pre-NACRT SMAI (low versus high; *P *=* *0.026), chemotherapy (reduced versus standard; *P *=* *0.036) and surgical procedure (pneumonectomy versus lobectomy or bilobectomy; *P *=* *0.037).

**Table 4:** Results of univariable and multivariable analyses for overall survival

Characteristics		Overall survival					
		Univariable analysis			Multivariable analysis		
		HR	95% CI	*P*-value	HR	95% CI	*P*-value
Age (years)	≥70	2.9	1.05–8.05	0.040			
Sex	Male	2.9	0.61–11.0	0.20			
Smoking	Smoker	2.3	0.53–9.67	0.27			
Pre-NACRT BMI	<22	1.4	0.60–3.06	0.46			
cStage	III	3.7	0.49–27.2	0.20			
FEV1.0%	<70%	0.6	0.24–1.56	0.31			
Chemotherapy	Reduced	2.3	0.26–5.29	0.062	2.6	1.06–6.30	0.036
SMAI (cm^2^/m^2^)	Low SMAI	2.5	1.09–5.50	0.029	2.5	1.12–5.63	0.026
Surgical procedure	Pneumonectomy	2.1	0.85–4.99	0.108	2.7	1.06–6.75	0.037
Perioperative bleeding (ml)	≥180 ml (median)	0.6	0.28–1.44	0.28			
Operating time (min)	≥350 min (median)	1.0	0.44–2.19	0.96			
Concomitant resection	Present	0.8	0.35–1.94	0.66			
Complications	Present	0.9	0.34–2.43	0.85			
pStage	≥II	0.9	0.40–2.01	0.80			

CI: confidence interval; cStage: clinical stage; FEV1: forced expiratory volume in 1 s; HR: hazard ratio; pStage: pathological stage; SMAI: skeletal muscle area index.

Supplementary Material, Table S1 presents the results of the univariable and multivariable analyses of the clinicopathological factors associated with DFS. In the univariable analyses, the significant prognostic factors for DFS were age (≥70 vs <70 years; *P *=* *0.003), and pre-NACRT SMAI (low versus high; *P *=* *0.045); in the multivariable analysis, only age (≥70 vs <70 years; *P *=* *0.003) was found to be the independent prognostic factor for DFS.

## DISCUSSION

This study revealed a significant association between low pre-NACRT SMAI and poor prognosis in patients with stage II/III NSCLC who underwent surgical resection after receiving NACRT. Patients with a low pre-NACRT SMAI had poor DFS and OS; furthermore, multivariable analysis identified low pre-NACRT SMAI as an independent predictor of OS.

Sarcopenia is caused by several factors such as ageing, physical inactivity, malnutrition and cachexia [4]. Patients with SMA-diagnosed sarcopenia have a worse prognosis for various types of cancers [12–14]. In the case of lung cancer, Suzuki *et al.* [5] demonstrated that sarcopenia correlates with poor outcomes in patients with completely resected early-stage NSCLC. Takamori *et al.* [9] reported that postoperative skeletal muscle loss correlates significantly with poor prognosis in patients with early NSCLC. Furthermore, Katsui *et al.* [15] elucidated that skeletal muscle mass correlates with prognosis in patients with stage III lung cancer who are receiving concurrent chemoradiotherapy. Consistent with the findings of aforementioned previous studies on lung cancer, our study demonstrated that sarcopenia with low SMA is a poor prognostic factor for OS in patients with stage II/III NSCLC who underwent surgical resection following NACRT.

Four reasons have been proposed to explain why patients with cancer with sarcopenia have a poor prognosis after surgery [16]. First, sarcopenia itself has a poor prognosis. Second, patients with sarcopenia tend to experience postoperative complications. Third, tumours in patients with sarcopenia have a higher malignant potential than those in those without sarcopenia. Fourth, patients with sarcopenia have lower levels of myokines. Furthermore, skeletal muscles have recently been identified as secretory organs producing myokines, which inhibit cancer cell growth and are involved in immune cell modulation [17, 18]. We identified no significant association of postoperative complications and pathological stage with low pre-NACRT SMAI. Therefore, we hypothesized that sarcopenia itself is associated with poor prognosis. Surgery after NACRT may be excessively invasive for patients with sarcopenia. The role of myokines has not been thoroughly explored yet; thus, further research is warranted to establish an association among skeletal muscle mass, myokines and postoperative outcomes in NSCLC.

Regarding the SMA measurement method using CT scans, Prado *et al.* [19] reported that the SMA in the 3rd lumbar vertebra (L3) is optimal for predicting prognosis in patients with cancer. Therefore, measuring the SMA in L3 is widely used to assess sarcopenia. However, in the treatment of lung cancer, L3 is not always included in the typical chest CT images. Thus, in thoracic diseases such as lung cancer, standards other than skeletal muscle mass at the L3 level are needed. Because Ishida *et al.* [20] showed a significant association between the cross-sectional area of the whole skeletal muscle mass at the L3 level and the erector spinae muscles at the Th12 level, the paravertebral SMA at the Th12 level may be used instead of the whole SMA at the L3 level to evaluate sarcopenia.

Because sarcopenia with a low SMAI can be assessed before NACRT, the patients may choose a less invasive alternative treatment, such as definitive chemoradiotherapy, rather than surgery after NACRT. However, evidence suggests that establishing a regular exercise and nutritional support program before surgery may increase daily calorie intake, protein intake, grip strength and skeletal muscle mass [21]. In high-risk patients, early intervention may increase skeletal muscle mass and lead to a better prognosis after surgery following NACRT.

Surgery following NACRT is a viable treatment strategy for locally advanced NSCLC. In this study, the 5-year OS rate for all patients was 72.5%, with a postoperative complication rate of 22.6%; no hospital deaths were reported. The 5-year OS rate was higher in our study than that of the inter-group trial 0139 (27.2%) [3]. One reason is that we included some patients with stage II NSCLC, which might have resulted in significant difference in the 5-year OS rates. Another reason is that concurrent chemoradiotherapy with S-1, including tegafur—an oral 5-fluorouracil prodrug—plus cisplatin, might have been effective. Yamaguchi *et al.* [8] studied the effect of using this induction treatment followed by surgery on patient survival and reported that the 3- and 5-year DFS rates in all 39 patients who underwent resection were 52.0% and 44.0%, respectively, whereas the 3- and 5-year OS rates were 77.4% and 61.7%, respectively. In the present study, 70% of patients also received chemoradiotherapy with the cisplatin plus S-1 regimen. Thus, surgery after NACRT appears to be a safe and effective treatment option for locally advanced NSCLC.

### Limitations

The present study has some limitations. This study was retrospective in nature, and the cohort size was small. In the statistical analysis, the cut-off value of SMAI was calculated using ROC analysis in this study, but the AUC did not indicate very good predictive accuracy, and this seemed due to the small number of patients. In addition, there were 6 patients with non-cancer-specific death in this study; then, SMAI might be a factor not only for lung cancer recurrence and death, but also for the risk of death from other causes. Moreover, surgery after NACRT is not the only treatment option for locally advanced NSCLC; the presence of an unmeasured bias in patient selection cannot be ruled out. Furthermore, muscle strength measurements that are necessary to assess sarcopenia could not be performed in this study. In the future, we would like to conduct a multicentre, prospective study to establish an accurate cut-off for SMAI and determine whether nutrition and exercise interventions actually improve sarcopenia and patient prognosis.

## CONCLUSION

In conclusion, our study demonstrated that low pre-NACRT SMAI is significantly associated with poor prognosis in patients with surgically resected NSCLC after NACRT. Furthermore, assessing sarcopenia based on pre-NACRT SMAI may be beneficial in determining optimal treatment strategies as well as suitable nutritional and exercise interventions.

## Supplementary Material

ivad020_Supplementary_Data

## Data Availability

The data underlying this article will be shared on reasonable request to the corresponding author.

## ABBREVIATIONS

BMI: Body mass index
CT: Computed tomography
DFS: Disease-free survival
L3: 3rd lumbar vertebra
NACRT: Neoadjuvant chemoradiotherapy
NSCLC: Non-small-cell lung cancer
OS: Overall survival
ROC: Receiver operating characteristic
S-1: Tegafur/gimeracil/oteracil
SMA: Skeletal muscle area
SMAI: Skeletal muscle area index
Th12: 12th thoracic vertebra

## References

[1] Fitzmaurice C , Akinyemiju TF, Al Lami FH, Alam T, Alizadeh-Navaei R, Allen C et al; Global Burden of Disease Cancer Collaboration. Global, regional, and national cancer incidence, mortality, years of life lost, years lived with disability, and disability-adjusted life-years for 29 cancer groups, 1990 to 2016: a systematic analysis for the global burden of disease study. JAMA Oncol 2018;4:1553–68. 29860482 10.1001/jamaoncol.2018.2706 PMC6248091

[2] Goldstraw P , Chansky K, Crowley J, Rami-Porta R, Asamura H, Eberhardt WE et al; International Association for the Study of Lung Cancer Staging and Prognostic Factors Committee Advisory Boards and Participating Institutions. The IASLC lung cancer staging project: proposals for revision of the TNM stage groupings in the forthcoming (eighth) edition of the TNM classification for lung cancer. J Thorac Oncol 2016;11:39–51. 26762738 10.1016/j.jtho.2015.09.009

[3] Albain KS , Swann RS, Rusch VW, Turrisi AT 3rd, Shepherd FA, Smith C et al Radiotherapy plus chemotherapy with or without surgical resection for stage III non-small-cell lung cancer: a phase III randomised controlled trial. Lancet 2009;374:379–86. 19632716 10.1016/S0140-6736(09)60737-6 PMC4407808

[4] Cruz-Jentoft AJ , Baeyens JP, Bauer JM, Boirie Y, Cederholm T, Landi F et al Sarcopenia: European consensus on definition and diagnosis. Age Ageing 2010;39:412–23. 20392703 10.1093/ageing/afq034 PMC2886201

[5] Suzuki Y , Okamoto T, Fujishita T, Katsura M, Akamine T, Takamori S et al Clinical implications of sarcopenia in patients undergoing complete resection for early non-small cell lung cancer. Lung Cancer 2016;101:92–7. 27794415 10.1016/j.lungcan.2016.08.007

[6] Degens JHRJ , Sanders KJC, de Jong EEC, Groen HJM, Smit EF, Aerts JG et al The prognostic value of early onset, CT derived loss of muscle and adipose tissue during chemotherapy in metastatic non-small cell lung cancer. Lung Cancer 2019;133:130–5. 31200819 10.1016/j.lungcan.2019.05.021

[7] Takada K , Yoneshima Y, Tanaka K, Okamoto I, Shimokawa M, Wakasu S et al Clinical impact of skeletal muscle area in patients with non-small cell lung cancer treated with anti-PD-1 inhibitors. J Cancer Res Clin Oncol 2020;146:1217–25. 32025867 10.1007/s00432-020-03146-5 PMC11804506

[8] Yamaguchi M , Toyokawa G, Ohba T, Sasaki T, Kometani T, Hamatake M et al Preoperative concurrent chemoradiotherapy of S-1/cisplatin for stage III non-small cell lung cancer. Ann Thorac Surg 2013;96:1783–9. 23998404 10.1016/j.athoracsur.2013.06.036

[9] Takamori S , Toyokawa G, Okamoto T, Shimokawa M, Kinoshita F, Kozuma Y et al Clinical impact and risk factors for skeletal muscle loss after complete resection of early non-small cell lung cancer. Ann Surg Oncol 2018;25:1229–36. 29327178 10.1245/s10434-017-6328-y

[10] Rutten IJ , van Dijk DP, Kruitwagen RF, Beets-Tan RG, Olde Damink SW, van Gorp T. Loss of skeletal muscle during neoadjuvant chemotherapy is related to decreased survival in ovarian cancer patients. J Cachexia Sarcopenia Muscle 2016;7:458–66. 27030813 10.1002/jcsm.12107 PMC4782251

[11] Heagerty PJ , Lumley T, Pepe MS. Time-dependent ROC curves for censored survival data and a diagnostic marker. Biometrics 2000;56:337–44. 10877287 10.1111/j.0006-341x.2000.00337.x

[12] Harimoto N , Yoshizumi T, Shimokawa M, Sakata K, Kimura K, Itoh S et al Sarcopenia is a poor prognostic factor following hepatic resection in patients aged 70 years and older with hepatocellular carcinoma. Hepatol Res 2016;46:1247–55. 26880049 10.1111/hepr.12674

[13] Villaseñor A , Ballard-Barbash R, Baumgartner K, Baumgartner R, Bernstein L, McTiernan A et al Prevalence and prognostic effect of sarcopenia in breast cancer survivors: the HEAL Study. J Cancer Surviv 2012;6:398–406. 23054848 10.1007/s11764-012-0234-x PMC3747827

[14] Levolger S , van Vugt JL, de Bruin RW, IJzermans JN. Systematic review of sarcopenia in patients operated on for gastrointestinal and hepatopancreatobiliary malignancies. Br J Surg 2015;102:1448–58. 26375617 10.1002/bjs.9893

[15] Katsui K , Ogata T, Sugiyama S, Yoshio K, Kuroda M, Hiraki T et al Sarcopenia is associated with poor prognosis after chemoradiotherapy in patients with stage III non-small-cell lung cancer: a retrospective analysis. Sci Rep 2021;11:11882. 34088965 10.1038/s41598-021-91449-z PMC8178326

[16] Kawaguchi Y , Hanaoka J, Ohshio Y, Okamoto K, Kaku R, Hayashi K et al Does sarcopenia affect postoperative short- and long-term outcomes in patients with lung cancer?—a systematic review and meta-analysis. J Thorac Dis 2021;13:1358–69. 33841929 10.21037/jtd-20-3072 PMC8024851

[17] Pedersen BK , Febbraio MA. Muscles, exercise and obesity: skeletal muscle as a secretory organ. Nat Rev Endocrinol 2012;8:457–65. 22473333 10.1038/nrendo.2012.49

[18] Benatti FB , Pedersen BK. Exercise as an anti-inflammatory therapy for rheumatic diseases—myokine regulation. Nat Rev Rheumatol 2015;11:86–97. 25422002 10.1038/nrrheum.2014.193

[19] Prado CM , Lieffers JR, McCargar LJ, Reiman T, Sawyer MB, Martin L et al Prevalence and clinical implications of sarcopenic obesity in patients with solid tumours of the respiratory and gastrointestinal tracts: a population-based study. Lancet Oncol 2008;9:629–35. 18539529 10.1016/S1470-2045(08)70153-0

[20] Ishida Y , Maeda K, Yamanaka Y, Matsuyama R, Kato R, Yamaguchi M et al Formula for the cross-sectional area of the muscles of the third lumbar vertebra level from the twelfth thoracic vertebra level slice on computed tomography. Geriatrics 2020;5:47. 32899577 10.3390/geriatrics5030047 PMC7555041

[21] Yamamoto K , Nagatsuma Y, Fukuda Y, Hirao M, Nishikawa K, Miyamoto A et al Effectiveness of a preoperative exercise and nutritional support program for elderly sarcopenic patients with gastric cancer. Gastric Cancer 2017;20:913–8. 28032232 10.1007/s10120-016-0683-4

