# Supplementary material for: Skeletal muscle area predicts the outcomes of non-small-cell lung cancer after trimodality therapy
Source: Interdiscip Cardiovasc Thorac Surg. 2023 Jan 25;36(2):ivad020. doi: 10.1093/icvts/ivad020 (PMC9901413; doi:10.1093/icvts/ivad020)
Supplement: ivad020_Supplementary_Data [file ivad020_supplementary_data.zip › Supplementary Table S1.docx]

**Supplementary Table 1**. Results of univariate and multivariate analyses of disease-free survival

|  |  | Disease-free survival | | | | | |
| --- | --- | --- | --- | --- | --- | --- | --- |
|  |  | Univariable analysis | | | Multivariable analysis | | |
| Characteristics |  | HR | 95% CI | *p*-value | HR | 95% CI | *p*-value |
| Age (years) | ≥70 | 2.7 | 1.42-5.33 | 0.0028 | 2.7 | 1.42-5.33 | 0.0028 |
| Sex | Male | 0.6 | 0.32-1.15 | 0.1233 |  |  |  |
| Smoking | Smoker | 0.9 | 0.41-1.89 | 0.7509 |  |  |  |
| cStage | III | 1.9 | 0.68-5.25 | 0.2258 |  |  |  |
| FEV 1.0% | <70% | 0.9 | 0.51-1.65 | 0.7788 |  |  |  |
| Chemotherapy | Reduced | 1.3 | 0.66-2.66 | 0.4268 |  |  |  |
| SMAI (cm^2^/m^2^) | Low SMAI | 1.8 | 1.01-3.15 | 0.0447 |  |  |  |
| Surgical procedure | Pneumonectomy | 1.2 | 0.56-2.55 | 0.6494 |  |  |  |
| Perioperative bleeding (mL) | ≥180mL(median) | 1.0 | 0.57-1.77 | 0.9861 |  |  |  |
| Operating time (minutes) | ≥350 min(median) | 1.5 | 0.83-2.60 | 0.1867 |  |  |  |
| Concomitant resection | Present | 1.1 | 0.63-2.02 | 0.6792 |  |  |  |
| Complications | Present | 1.5 | 0.81-2.83 | 0.1904 |  |  |  |
| pStage | ≥II | 1.8 | 0.96-3.21 | 0.0655 |  |  |  |

cStage, clinical stage; FEV1, forced expiratory volume in 1 second; SMAI, skeletal muscle area index; HR, hazard ratio; CI, confidence interval; pStage, pathological stage
