# Supplementary figures and images for: Skeletal muscle area predicts the outcomes of non-small-cell lung cancer after trimodality therapy
Source: Interdiscip Cardiovasc Thorac Surg. 2023 Jan 25;36(2):ivad020. doi: 10.1093/icvts/ivad020 (PMC9901413; doi:10.1093/icvts/ivad020)

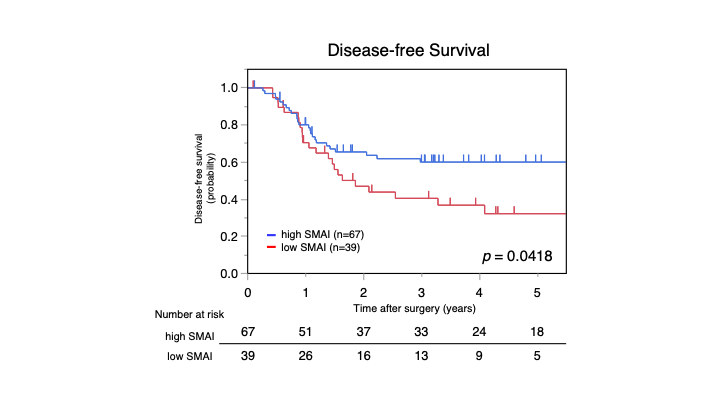

Supplement: ivad020_Supplementary_Data [file ivad020_supplementary_data.zip › Supplementary FigureS1.tiff]
